# Enhancing engineering ethics education (EEE) for green intelligent manufacturing: Implementation performance evaluation of core mechanism of green intelligence EEE

**DOI:** 10.3389/fpsyg.2022.926133

**Published:** 2022-07-27

**Authors:** Shi Yin, Nan Zhang

**Affiliations:** ^1^College of Economics and Management, Hebei Agricultural University, Baoding, China; ^2^School of Marxism, Hebei Agricultural University, Baoding, China

**Keywords:** green intelligence, engineering education, ethical education, fuzzy analytic hierarchy process, performance evaluation

## Abstract

The characteristics of green intelligent (GI) engineering ethics emphasize the necessity of GI engineering ethics education (EEE). The ethics education of GI engineering is in the development stage, and it is urgent to fully understand the significance of evaluating the development of GI EEE. Only based on the GI manufacturing situation system to understand the implementation status of the core education of EEE can we objectively grasp the improvement space of GI EEE. In this study, the corresponding indicators were selected from three dimensions of cultivation education, collaborative education, and situational education to form the element community of evaluation indicators. The fuzzy analytic hierarchy process and the fuzzy comprehensive evaluation method were used to empirically evaluate the implementation of the key mechanism of GI EEE. The results are as follows. (1) The key education of GI EEE includes cultivation education of micro dimension, collaborative education of medium dimension, and situational education of macro dimension. (2) The most important education is to strengthen the ethics education of GI engineering in the training process of college students. The coordination of GI EEE is becoming more and more important, and the integration and construction are the important pursuit of GI EEE. (3) The cultivation education, collaborative education, and situational education of GI EEE are all at a general level. (4) There is not only a gap between theory and practice in GI EEE but also insufficient attention to localization and coordination issues. The willingness of the government to participate in the ethical education of GI engineering is very insufficient. The optimized space of training education includes teaching cases and full-cycle ethical education.

## Introduction

In recent years, various engineering accidents occur frequently, and many engineering technologies, such as transgenic technology, embryo technology, and artificial intelligence, have generated more and more ethical disputes (Yin et al., 2020). There are also multiple ethical risks in engineering practice, such as the environmental risks brought by the integration of various technologies and their application to nature, and the quality and safety risks of using technologies to build artifacts (Martin et al., 2021). Due to the technical complexity and social connection of engineering itself, engineering ethics is no longer a problem within engineering but related to the survival and development of the whole human society. Mote, a member of the US National Academy of Engineering, pointed out that engineering in the twenty-first century is a combination of the technology part “creation and solution” and the user part “people and society.” It is this ethical load, including conscience and benevolence, that constitutes the ultimate goal and value of engineering and promotes its continuous development (Yin et al., 2020). The inherent nature of the uncertainty in the development of science and technology innovation will lead to more challenging ethical problems in the future. The emergence of engineering ethics problems is often caused by the lack of ethical awareness, the insufficient estimation of the consequences of engineering activities, the conflict of interests of all parties in the project, the weak consciousness of natural social responsibility, and other factors, which all come from the subject of engineering practice (Yin and Zhang, 2021). Engineers who are direct participants in engineering practice often face ethical dilemmas, and the work they do, such as designing, planning, and managing infrastructure, as well as designing materials and systems, involves risk (Trentesaux and Karnouskos, 2022). Engineers, therefore, have a high degree of responsibility to society and stakeholders. It is necessary to strengthen students' understanding of engineering ethics in the teaching process so that they can have a certain sense of ethics after engaging in information related work, and can use the learned ethical knowledge to analyze, judge, and make decisions on their engineering practice (Hsu, 2020).

With the acceleration of the new technological revolution characterized by “green and intelligent,” the integration of green manufacturing and intelligent manufacturing has become the key to the high-quality development of manufacturing engineering. Intelligent manufacturing and green manufacturing have become two major development directions of contemporary manufacturing engineering (Popescu et al., 2020). Although both serve the manufacturing process, intelligent manufacturing and green manufacturing have different manufacturing concepts and priorities. The intelligent manufacturing mode focuses on how to use information flow and data flow in the manufacturing process to endue manufacturing system with intelligence, thus improving production efficiency and reducing operating costs. Green manufacturing focuses on how to plan the material flow and energy flow in the manufacturing process to improve the resource utilization rate and green production efficiency of the manufacturing system. This then coordinates the economic benefit and the social benefit of the enterprise. In actual production, intelligent manufacturing and green manufacturing have synergism and complementarity. Intelligent manufacturing and green manufacturing are two subsystems belonging to manufacturing system from the point of view of system goal synergy (Yin et al., 2022a). From the perspective of system function complementarity, the intelligence brought by intelligent manufacturing subsystem is conducive to the rational planning and utilization of resources of the green manufacturing subsystem. The low carbonization insisted by the green manufacturing subsystem is a necessary condition for the intelligent manufacturing subsystem to reduce cost and improve efficiency. The key to the integration of green manufacturing and intelligent manufacturing lies in the continuous innovation and promotion of important green intelligent (GI) key generic technologies in the field of industrial engineering (He and Bai, 2021). Intelligent manufacturing endows manufacturing system with new functions of self-organization, self-regulation, and self-operation production intelligence. Green manufacturing carries out dynamic planning of material flow and energy flow for product research and development, production, maintenance, recycling, and other manufacturing processes to maximize green production efficiency in the manufacturing process. In the process of GI manufacturing, the parallel engineering and integration engineering of the intelligent and green manufacturing process should be established to achieve the dual goals of improving production efficiency and realizing cleaner production (He and Bai, 2021). With the remolding of traditional manufacturing engineering by GI manufacturing, new requirements of engineering ethics also appear.

GI engineering ethics is the standard and guidance for engineers and engineering activities, and its connotation includes two aspects. On the one hand, the pursuit of goal value is the commitment of engineering and engineers to human progress and the pursuit of improving human wellbeing, which is also the basis of engineering ethics (Trentesaux and Caillaud, 2020). The other side contains various engineering ethics rules and norms. These specific systems of ethical principles influence the way project stakeholders live, how decisions are made, and how those decisions later affect human society. Although the innovation and rapid development of GI manufacturing technology and its application in engineering will bring great contributions to the welfare of human society subjectively, it will inevitably pose severe challenges to human ethics (Iphofen and Kritikos, 2021). This forms an increasingly sharp ethical problem of GI engineering. The problems mainly include those caused by the use of information technology and artificial intelligence. The wide application of information technology in the field of engineering will impact the boundary of traditional moral responsibility, thus causing ethical problems that must be paid great attention to. The problems include the possible infringement of copyright owners of various works caused by digitization and Internet, the possible privacy protection problems caused by the unrestrained dissemination and collection of massive information to the public, and the abandonment of traditional social ethical life by addicts in the virtual network world (Royakkers et al., 2018). Improper use of artificial intelligence technology may pose a great threat to human society and trigger new ethical issues. For example, robots challenge, threaten, and harm humans. Humanoid robots impact human life and its way, and challenge law and public order. Brain-computer interface technology and face recognition technology bring personal privacy issues.

Compared with traditional engineering, GI engineering has the typical characteristics of complexity, integration, socialization, and globalization. GI engineering projects must not only address technical and economic issues but also ethical issues related to safety, cost-effectiveness, resources, the environment, and ecology (Naphan-Kingery et al., 2019). The characteristics of GI engineering ethics are mainly shown in the following three aspects. (1) The development speed of digital technology and the transmission speed of information are incomparable to many traditional engineering technologies. At the same time, because the transmission speed is beyond imagination, the spread of good in the society makes people happy, while the spread of evil makes people unprepared (Burr et al., 2020). As the development of engineering ethics often lags behind the development of technology, the researchers and educators of engineering ethics feel more pressure in the face of a large number of emerging and rapidly spreading new phenomena and new events today. (2) In the digital society, more information is generated through production and exchange, and is flooded in all spaces of the society. At the same time, people are able to record more and more information, and even to present historical events in front of their eyes, which further expands the concept of time and space. It is a great challenge to carry out engineering ethics research and education in such a wide range of fields. (3) The above two characteristics result in the complexity and diversity of engineering ethics research in the field of information. This means that ethical research and education should not only consider the impact of engineering technology but also consider the human thought and spirit it carries (Sorenson, 2019).

The ethical problems and characteristics of GI engineering highlight the necessity of ethics education oriented to GI engineering. As a part of quality education, GI engineering ethics education (EEE) helps students to pursue the value rationality of science and technology in future engineering practice (Balakrishnan et al., 2021). It is responsible for the sustainable development of human society in the future, and deals with the relationship between engineering and humans, society and nature objectively, fairly and impartially (Frigo et al., 2021). This maximizes the positive impact of engineering in promoting human safety, health, and wellbeing.

The core of EEE for GI manufacturing is to guide students to deal with the ethical problems in engineering independently. This requires students to establish a conscious sense of responsibility for the overall social significance and long-term social impact of engineering activities, and have the practical ability to identify, analyze, and solve new problems in engineering ethics (Stransky et al., 2021). On the basis of a dialogue with the public and other stakeholders, engineering ethics is constructed to make their ethical decisions and actions have practical effects on engineering practice. At present, the ethics education of GI engineering is in the development stage. It is urgent to fully understand the significance of evaluating the development of GI EEE and to promote teaching through evaluation. Only based on the GI manufacturing situation system to understand the implementation status of the core education of EEE can we objectively grasp the improvement space of GI EEE. Therefore, it is of great theoretical and practical significance to establish an effective evaluation index system and assign weight to each index to evaluate the implementation status of the core education of GI EEE.

In 1980, Hastings Center put forward a five-point consensus on the goal of ethical education, namely, stimulating ethical imagination, identifying ethical problems, analyzing key ethical concepts and principles, helping students to deal with ambiguous problems and improving the responsibility of the educated (Avci, 2021). Many scholars analyzed the teaching objectives of engineering ethics courses in colleges and universities and summarized a list of EEE objectives, including nine items (Balakrishnan et al., 2021; Frigo et al., 2021). These contents include ethical imagination, students' discovery of problems, students' analysis of key ethical concepts and principles, students' handling of ambiguity, students' serious treatment of ethical issues, students' sensitivity to ethical issues, students' mastery of relevant ethical principles, ethical judgments and ethical will. Newberry (2004) proposed three categories of educational goals: emotional participation (willingness to make ethical decisions), intellectual participation (using ethical decision-making tools to solve ethical problems), and specialized knowledge (being familiar with ethical concepts, theories, and norms) (Newberry, 2004). The above contents can be further transformed into four aspects of EEE goals. (a) Enhanced ethical sensitivity (identification of ethical issues); (b) Development of ethical knowledge (understanding of terminology, ethical codes, ethical theories); (c) Strengthening ethical judgment (making judgments and decisions based on sound grounds rather than chance or common sense); (d) Enhancing ethical commitment, confidence, and courage (taking action to address ethical issues). Based on the research results, this study fully absorbs the viewpoints of the above scholars and integrates the objectives of EEE into the following three aspects. (1) Ethical sensitivity or awareness. The educational objective focuses on enhancing students' awareness of ethical situations. It is necessary to improve the students' sensitivity to the ethical issues they may encounter after working in the industry, and to understand the issues related to people, society, and nature behind engineering technology (Maxwell et al., 2021). (2) Ethical knowledge and skills. The educational goal focuses on helping students know how to avoid and solve ethical problems involving honesty, fairness, wellbeing, environmental protection, and war. This goal can be achieved in a variety of ways, such as learning ethical principles, ethical theories, classic cases or carrying out engineering practice (Saada, 2022). (3) Ethical willpower. The educational goal focuses on internalization and requires students to have confidence and courage in dealing with ethical issues. On the basis of understanding and mastering the values of the engineer community (such as justice and sustainable development, etc.), the engineer should form and develop individual moral laws, be able to judge right and wrong autonomously, and achieve the unity of knowledge and action (Balakrishnan et al., 2020).

The teaching strategies of engineering ethics mainly include course strategies, teaching contents, teaching methods, and examination methods. At present, the main curriculum strategy includes an independent curriculum, an embedded curriculum, and integration with a non-technical curriculum (Mitcham and Englehardt, 2019). (1) Independent courses. Independent courses, the most common form of instructions, are usually taught as electives by regular teachers over a full semester, and the syllabus covers a variety of ethical topics, such as engineering ethics and politics (Bielefeldt et al., 2018). (2) Embedded courses. The embedded curriculum emphasizes the introduction of ethics education in all professional courses and the separation of knowledge points of ethics education into different professional courses, which is one of the main trends of current ethics education (Grosz et al., 2019). (3) Integration of engineering ethics and non-technical courses. The EEE should be integrated into non-technical courses of humanities and social sciences, especially science, technology, and society courses to carry out EEE (Winberg et al., 2020). In addition, the delivery strategy mainly consists of a summit course (incorporating engineering ethics into the final project) and a seminar for senior students. The teaching content of engineering ethics mainly includes the following aspects (Haghighattalab et al., 2019; Martin et al., 2021). (1) Cases. Common engineering ethics cases include challenger launch failure and Bhopal chemical leakage, etc. Cases are mainly from real historical events or fictional scenarios. (2) Ethical code. (3) Ethical dilemmas or conflicts of interest. (4) Ethical theory, mainly including deontology, utilitarianism, morality ethics, China will also teach the theory of Confucianism, Taoism, and other schools. (5) Commonly used concepts of engineering ethics, ethical decision-making tools, engineering and laws and regulations (especially intellectual property rights), engineering and sustainable development are also frequently taught. In addition, China also attaches importance to the teaching of craftsman spirit, excellent traditional culture, patriotism, and model worker spirit in EEE. At present, there is no significant difference in the teaching methods advocated by different scholars.

Teaching methods include case studies, group or classroom discussions, guided teaching, literature learning, project-based learning, games or role playing, service learning, etc. (Balakrishnan et al., 2020, 2021; Martin et al., 2021). In addition, engineering ethics educators have also explored and applied other new teaching methods. (1) Case studies. Case study is one of the most common methods in EEE. Cases provide an effective medium for examining ethical dilemmas from multiple perspectives and encourage students to develop action plans based on different ethical theories so that they can simulate ethical decisions in a professional context as realistically as possible. (2) Games and role playing. Through games, students can gain a practical understanding of ethical dilemmas. In the games, the students can fully exercise and improve the ability of negotiation, strategic planning, speech, and so on. (3) Service learning. At present, service learning is paid more and more attention in EEE. This method requires students to integrate into the real world of engineering ethics and experience and outline the real state of engineering ethics through participating in a community service project. In service learning, students participate in organized and continuous service activities related to course learning and meet specific needs of the community. Then, the students summarize and explain the experience through classroom discussions or diaries. Service learning is widely used in education and teaching of many subjects, and its popularity continues to grow.

The assessment methods of engineering ethics courses are as follows (Hess and Fore, 2018; Hagendorff, 2020). (a) Written reports, usually completed by individuals or groups; (b) Presentation; (c) exams/in-class tests; (d) Literature reading; (e) Daily performance, such as attendance, online and offline interactions, group performance, study notes, etc.; (f) Creative product production, such as the development of environmental protection products that can effectively solve the problem of sewage discharge.

The evaluation of the effect of EEE in the United States has the following characteristics (Hess and Fore, 2018). (1) the evaluation focuses on the ethical reasoning ability of engineering students; (2) Formed a relatively complete and scientific evaluation tool system for EEE; (3) Comprehensive use of quantitative and qualitative evaluation methods; (4) Limitations of the study design were noted; (5) Pay attention to the diversity of the assessment subject and assessment environment. Compared with foreign countries, China is still in the exploratory stage in the determination of evaluation subjects, the exploration of evaluation methods, and the formulation of evaluation tools. At present, EEE in China has been grounded in some colleges and achieved initial results. However, it is urgent to fully understand the significance of evaluation to the development of GI EEE and anchor the actual needs of new engineering construction (Ye et al., 2020). To explore the evaluation subjects, methods and tools suitable for different development stages of EEE are beneficial to promote the high-quality development of EEE in China. At present, the effect evaluation of EEE mainly uses qualitative evaluation and quantitative evaluation (Bielefeldt et al., 2018). (1) Quantitative evaluation. The most common quantitative assessment methods mainly include questionnaires, pre/post tests, student productivity, moral assessment tools, etc. (2) Qualitative evaluation. The most common qualitative assessment methods mainly include interviews or focus groups and classroom observations by teachers. Therefore, a combination of qualitative and quantitative assessment methods can be used to assess the comprehensive level of core mechanism of GI EEE.

Based on the existing research, it is summarized as follows. First, the goal of EEE is clear, and it is a unity formed by the superposition of feeling, knowing, and expressing. Second, the teaching strategies of EEE are rich and diversified. Thirdly, the effect of EEE is affected by many factors. Fourthly, evaluation feedback is an important part of quality control and continuous improvement in EEE. With the gradual maturity of EEE teaching methods, EEE evaluation is regarded as an important means to promote the in-depth development of EEE practice. Its purpose is to investigate whether all kinds of EEE teaching models are effective and to pay attention to whether students meet some educational goals. The evaluation object is no longer limited to students but includes teachers, teaching materials, teaching environment, and other categories. In recent years, scholars have gradually attached importance to the evaluation of the process and results of GI EEE, and emphasized the continuous optimization of teaching programs to maintain their effectiveness. However, compared with developed countries, there are few practical exploration and theoretical research achievements on the evaluation of GI EEE in China.

At present, the integration of green manufacturing and intelligent manufacturing has become the key to high-quality economic development. The improvement space of GI EEE can be dialectically grasped only by understanding the implementation status of key education of EEE based on GI manufacturing situation system. Therefore, it is very necessary to establish an effective evaluation index system and assign weight to each index to evaluate the implementation status of key education of GI EEE. On the one hand, the theoretical structure of the key education of GI EEE is analyzed and discussed. On the other hand, by constructing the evaluation index system, the paper conducts an evaluation survey for multiple types of personnel, and empirically evaluates the implementation status of key education of GI EEE in colleges and universities by using a fuzzy analytic hierarchy process and a fuzzy comprehensive evaluation method. It is helpful to find the educational obstacles affecting the development of GI EEE on the basis of rational reflection of objective facts. This is conducive to focusing on the key and difficult points and weak links, and observing the overall trend of generation, change, and iteration of GI EEE.

The rest of this paper is as follows. Section Core Mechanism and Evaluation System is a core mechanism and an evaluation system. Fuzzy AHP and a fuzzy comprehensive evaluation method are shown in section Methodology. Section Results and Discussion is the results of fuzzy comprehensive evaluation of three layers. Conclusions and future prospects are presented in section Conclusions.

## Core mechanism and evaluation system

### GI EEE

The ethical education of GI engineering is influenced by many factors (intelligent engineering characteristics, educational consensus, carbon reduction, and efficiency increase) (Holsapple et al., 2012; Ngoepe et al., 2022). Situational education, collaborative education, and cultivation education play their roles from different angles. Finally, the effect is to enhance the quality of college education supply and gather the synergy of ethical education. If the above three kinds of education are in a positive state, it will make EEE run smoothly and orderly, and ethics teaching can also better enter the ear, the brain, and the heart. In order to further explore the inner relationship of the key education of GI EEE, the conditional matrix tool was used to analyze situational education, collaborative education, and cultivation education. The conditional matrix divides elements such as conditions and consequences into many levels from micro to macro, including: (1) Action; (2) Interaction; (3) Collective; (4) Secondary organization; (5) Organization and system; (6) Community; (7) Country; (8) International.

**Figure 1:**
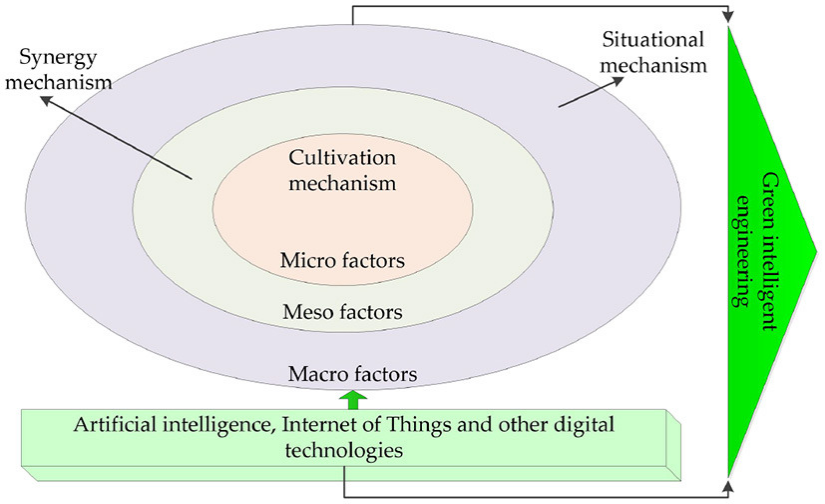
A core mechanism of GI EEE.

As shown in Figure 1, the key education of GI EEE includes: cultivation education of micro dimension, coordination education of medium dimension, and situational education of macro dimension.

(1) Cultivation and education. In training and education, educators transform the GI ethical concepts based on the country, society, and class to the educated by means of certain means and methods. To meet the requirements of GI engineering ethical literacy, training education is an inevitable measure of GI EEE (Zhang et al., 2022). Training education is an inevitable measure of GI EEE. The cultivation education responds to the problem of how to enhance the supply quality of the educational end of colleges and universities through GI EEE. The practice of GI EEE has not yet effectively responded to the core knowledge system, teaching syllabus, education methods, and other key issues.

(2) Collaborative education. Collaborative education is that all intermediary objects participating in GI EEE activities are combined into an orderly structure and function whole under the guidance and restriction of certain rules, so as to strengthen the practical efficiency of EEE. Collaborative education is the objective choice of GI EEE. Collaborative education responds to the question of how to gather the collaborative power of ethical education in GI EEE (Young et al., 2021). At present, the concept of collaborative development has become an important proposition for the development of higher education. In the collaborative perspective, education emphasizes the interactive connection among all elements, especially the dynamic resource sharing, and realizes the common development goals by means of the optimal allocation of information and resources. The development of GI EEE needs the synergistic effect of all intermediary objects, and cannot only rely on the initiative of universities. GI EEE is a complex and systematic project, which needs to give play to the leading role of the government and strengthen the incentive and support of the system. This also needs to give play to the advantages of enterprise organizations, promote school-enterprise cooperation, and encourage enterprises to take the initiative to undertake the responsibility of EEE for employees (Kim, 2022).

(3) Situational education. The comprehensive description of GI EEE cannot be separated from the systematic interpretation of situational education. The ethics education of GI engineering in developed countries has different characteristics. Can the region copy the experience of other countries? How to do the local ethics education of GI engineering well? The answers to the above problems lie in the fields constructed by situational education, including social factors, natural factors, and spiritual factors (Zhang and Zhu, 2021). Situational education is an inevitable requirement of GI EEE. The field of GI EEE integrates the needs and value judgment of things, and reflects the setting of identity and position of things, the choice of educational topics and contents, and the trend of teaching practice. The correct choice of each region is not only to introduce and learn the GI EEE from developed countries but also to highlight the nationality and identify the heterogeneous characteristics that cannot be perfectly explained by the GI EEE theory (Maqsoom et al., 2020). In addition, each region should try to develop regional characteristics of EEE, and finally put forward the local EEE program.

### Evaluation system of implementation effect

GI EEE is the product of the unity, integration, influence, and deployment of various elements in key education, and also the result of the comprehensive effects of training education, collaborative education, and situational education. Corresponding indexes are selected from the three dimensions of cultivation education, collaborative education, and situational education to form the element community of evaluation indexes. To ensure the scientific nature and accuracy of index selection, Harbin Engineering University, Yanshan University, long-term commitment to EEE, engineering education and engineering ethics research of three GI engineering ethics teachers, education economics and management professors, and three science and technology philosophy and ethics professors was invited to set an index and the statement for further revision. Through repeated communication, 3 first-level indicators, 8 second-level indicators, and 27 third-level indicators were finally determined. Evaluation system of implementation effect is shown in Table 1.

**Table 1:** Evaluation system of implementation effect.

**Main criterion layer**	**Sub-criteria layer**	**Scheme layer serial**	**Number**	**Interpretative statement**
Training and education A	Faculty A1	Teachers' professional level and ability A11	1	The professional level and ability of teachers are competent for the work of GI ethics education
		Number and structure of teaching staff A12	2	Building a reasonably structured teaching staff
		Teacher career development opportunities A13	3	Teachers can get full career development opportunities
	Teaching process A2	Course material A21	4	It can play a basic role in guiding the direction of GI EEE and ensuring the high-quality development of education.
		Teaching case A22	5	It can provide rich and diverse case choices for relevant teachers to better support the realization of the goal of GI EEE.
		Practice resource A23	6	It can bring students real ethical problems of GI engineering and improve their ability to deal with ethical problems of GI engineering.
	Teaching quality A3	Combination of theory and practice A31	7	It can help students understand the ethical issues of GI engineering and the ethical norms and norms of GI engineering
		Full cycle ethics education A32	8	Organically run through professional courses, graduation design and other links
		Course evaluation and improvement A33	9	Timely evaluate and improve the GI engineering ethics course
Collaborative education B	Government B1	Supporting system construction B11	10	The government actively promotes the construction of supporting system for ethics education of GI engineering
		Attention at the policy level B12	11	The government emphasizes the value of GI EEE from the perspective of policy
		Funding level B13	12	Various research topics or educational reform projects have fully supported the research and practice of GI EEE
	Enterprise organization B2	Attach importance to talent ethical literacy B21	13	Enterprises (especially engineering enterprises) pay full attention to the green intelligence and ethical literacy of talents
		Participation form and quality B22	14	Schools and enterprises jointly formulate the training objectives and training programs of GI EEE, jointly develop ethics courses, and provide teaching practice resources
		Willingness to participate B23	15	Enterprises have a strong willingness to participate in GI EEE
Situational education C	International vision C1	Grasp of Global Frontier Situation C11	16	Learn and introduce foreign advanced experience and teaching resources
		Academic dialogue C12	17	Published research articles on GI EEE in foreign journals
		Practice dialogue C13	18	Cooperate with foreign universities to set up GI ethics courses
	Regional discourse C2	Theoretical localization C21	19	Constructing educational theory in line with national conditions and the characteristics of GI engineering
		Practice localization C22	20	Combined with the specific situation, carry out GI ethics innovation on the educational concept, orientation, content and methods
		Local demands capture C23	21	In the process of carrying out GI EEE, the regional cultural background, social system and engineering practice have been given full attention

(1) Training and education. Teachers' personal experience, professional experience, and belief dynamics affect teachers' identity, and then affect teachers' effectiveness and practice in the classroom. Teachers' teaching is influenced by many factors from inside and outside the classroom, such as professional knowledge, subject background, and teaching content (Lomask et al., 2018). Teachers' Colleges (departments), gender, teaching years, practical experience of GI engineering, and adopted curriculum delivery strategies (the independent curriculum, the embedded curriculum, integration with the non-technical curriculum, the vertex curriculum, etc.) all have an impact on teaching effectiveness. The strength of teachers not only determines the quality of GI engineering ethics teaching but also determines whether the concept of GI EEE can be implemented This mainly includes the number of GI engineering ethics teachers and the interdisciplinary teacher team. Strengthening interdisciplinary cooperation with humanities and social sciences departments is an important link for engineering departments to deeply understand and grasp the specific ethical issues in the field of GI engineering. There is a shortage of teachers who understand GI engineering and can teach ethics courses, which requires inviting part-time teachers from government, enterprises, and other institutions to teach as much as possible. Funding resources from universities and governments are an important support to continuously promote teachers' innovative educational practice. Teachers' career development opportunities also include the adequacy of teachers' access to teacher training opportunities (Lawlor, 2021). GI EEE must strengthen the construction of teaching resources of GI EEE. GI EEE provides sufficient education and teaching resources, including curriculum materials, teaching cases, and practice bases. The construction of teaching materials for GI engineering ethics needs to be based on scientific research. The construction of the GI engineering ethics case base is a comprehensive work. The reason why an advanced learning concept is suitable for GI EEE is that the thinking characteristics of different cognitive development stages are different. In educational practice, the corresponding educational focus and operation strategies should also be different at each stage; appropriate measures are taken to complete key tasks, which can make the educational effect work with half the effort (Lavi and Dori, 2019).

(2) Collaborative education. For the collaborative goals of GI EEE, on the one hand, the all-round development of GI EEE should be actively supported. This is not only conducive to promoting the multi-angle integration and sharing of educational resources but also can form a huge resource supply source. On the other hand, it is necessary to promote the independent development of multiple governance subjects, including the government and enterprises and enhance the consciousness of conscious participation (Borenstein et al., 2019). The gradual development of long-term interests involves the foundation for independent development and enhancing communication and interaction. On the basis of mutual benefits and win-win, the collaborative promotion of GI EEE should be carried out. In terms of promoting goals, we should focus on realizing strategic coordination and construction coordination, and take co-construction and sharing as the core content. In the standardization of teaching system, GI engineering ethics, joint construction of university teaching resources, teacher training, and other aspects of cooperation and exchange of needs. The normalization and standardization of multi-subject participation are important foundations in the overall development of GI EEE. According to the theory of synergy advantage, member structure is dynamic. At present, enterprises have not been deeply involved in the collaborative development of GI EEE, and the auxiliary characteristics at the edge are obvious (Vveinhardt et al., 2019). However, as the main employment channel for engineering students, enterprises play a special role in the education of GI engineering ethics. In order to realize the organic combination of GI engineering ethics and career, some colleges and universities began to explore ways to cooperate with enterprises to carry out GI EEE. Enterprises are becoming an important part of the coordinated development of GI EEE. Some universities use school-enterprise cooperation platforms to let students go deep into the grassroots of enterprises (Zhang and Zhu, 2021). Students communicate with GI engineers of enterprises, and constantly strengthen the ethics of GI engineering in the process of verifying and correcting their own career.

(3) Situational education. Modern GI engineering projects not only involve technical and economic issues but also are related to sustainability, security, cost effectiveness, resources, ecological environment, and other issues (Polmear et al., 2021). The impact of automation and robot technology on human psychology, the impact of information technology on human society, and the threat of genetic engineering on human dignity are becoming new topics in the ethical education of GI engineering. GI EEE in China is undergoing a process from transplantation, imitation, verification to transformation and integration (Clancy, 2021). However, the duration is relatively short, and the real absorption and internalization are less. The depth and breadth should be expanded. Therefore, system understanding and system integration of system construction are very lacking. To give full play to the energy and function of GI EEE, it should be endowed with the ability of self-perfection and development. The localization of ethical education system construction of GI engineering focuses on two ways. First, the local transformation of professionalism has gradually established the GI engineering ethical standard system and the GI engineering vocational system construction theory under the guidance of discourse. The acceptance of GI EEE assessment will be a necessary prerequisite for the certification of professional engineers in the future. Secondly, the certification of GI engineering education based on real needs should clarify the requirements that engineering graduates must possess the ethical literacy of GI engineering.

## Methodology

### Determination of assessment methods

In the study, the evaluation system of implementation effect includes the main criterion layer, the sub-criteria layer, and the scheme layer serial. The evaluation of GI EEE implementation status is a typical multi-factor comprehensive evaluation problem. Most of the indicators are qualitative indicators with fuzzy characteristics, which are difficult to accurately judge and grade. The fuzzy comprehensive evaluation method is a kind of comprehensive evaluation method based on fuzzy mathematics. The comprehensive evaluation method transforms qualitative evaluation into quantitative evaluation according to the membership degree theory of fuzzy mathematics. Fuzzy mathematics makes an overall evaluation of things or objects restricted by many factors. It has the characteristics of clear results and strong systematicness, and can solve fuzzy and difficult to quantify problems well. Analytic hierarchy process (AHP) can clearly show the attribute weight of indicators at each level (Yin et al., 2022b). The weight is determined by experts based on triangular fuzzy numbers. The advantages of triangular fuzzy numbers are as follows: First, the expert team is very familiar with the field of triangulated fuzzy books. Secondly, the triangle fuzzy number can solve the contradiction that the performance of the evaluated object cannot be measured accurately but can only be evaluated by natural language. AHP is an effective tool to deal with complex decision problems. In view of the uncertainty caused by expert subjective judgment and language fuzziness in the use of AHP, the traditional nine-level scale method is combined with triangular fuzzy number. In the process of using the fuzzy AHP method, the step of the consistency test can be omitted (Buckley, 1985; Akkaya et al., 2015). Therefore, the fuzzy AHP method and the fuzzy comprehensive evaluation method based on the triangular fuzzy number were used to assess the comprehensive level of the core mechanism of GI EEE.

### Fuzzy AHP

Based on this combination, the established nine-level fuzzy scale is shown in Table 2.

**Table 2:** Expert judgment term and fuzzy number transformation relationship.

**Scale**	**Meaning**	**Triangular fuzzy number**	**Reciprocal**
1	The two elements are equally important	(1,1,1)	(1,1,1)
2	Between equally important and slightly important	(1,2,3)	(1/3,1/2,1)
3	The former is slightly more important than the latter	(2,3,4)	(1/4,1/3,1/2)
4	Between slightly important and more important	(3,4,5)	(1/5,1/4,1/3)
5	The former is stronger and more important than the latter	(4,5,6)	(1/6,1/5,1/4)
6	Between strong importance and strong importance	(5,6,7)	(1/7,1/6,1/5)
7	The former is more important than the latter	(6,7,8)	(1/8,1/7,1/6)
8	Between strong importance and extreme importance	(7,8,9)	(1/9,1/8,1/7)
9	The former is more important than the latter	(8,9,9)	(1/9,1/9,1/8)

Table 2 was used to collect experts' judgment opinions on the relative importance of each factor. Assuming that there are *n* risk factors in an indicator layer, and ${ã}_{ij}^{\left(k\right)}$ is the relative importance of the *i* factor judged by the *k* expert to the *j* factor, the fuzzy judgment matrix **Ã**^(*k*)^ of this indicator layer is shown in Formula (1).


(1) $$\text{​​}\begin{array}{l} A^{\left(k\right)}=\left({\tilde{a}}_{ij}^{\left(k\right)}\right)=\\ \left[\begin{array}{llll} \left(1,1,1\right) & \left(l_{12}^{\left(k\right)},m_{12}^{\left(k\right)},u_{12}^{\left(k\right)}\right) & \cdots & \left(l_{1n}^{\left(k\right)},m_{1n}^{\left(k\right)},u_{1n}^{\left(k\right)}\right)\\ \left(1/u_{12}^{\left(k\right)},1/m_{12}^{\left(k\right)},1/l_{12}^{\left(k\right)}\right) & \left(1,1,1\right) & \ldots & \left(l_{2n}^{\left(k\right)},m_{2n}^{\left(k\right)},u_{2n}^{\left(k\right)}\right)\\ \vdots & \vdots & \vdots & \vdots \\ \left(1/u_{1n}^{\left(k\right)},1/m_{1n}^{\left(k\right)},1/l_{1n}^{\left(k\right)}\right) & \left(1/u_{2n}^{\left(k\right)},1/m_{2n}^{\left(k\right)},1/l_{2n}^{\left(k\right)}\right) & \cdots & \left(1,1,1\right) \end{array}\right]\;\\ \;k=1,2,\cdots ,K,\;i,j=1,2,\cdots ,n \end{array}$$

Then the following modified formula is used to calculate the triangular fuzzy number of the weight of risk factors at each level.


(2) $$\begin{array}{l} {\tilde{S}}_{i}^{\left(k\right)}=(\frac{\sum_{j=1}^{n}l_{ij}^{\left(k\right)}}{\sum_{j=1}^{n}l_{ij}^{\left(k\right)}+\sum_{z=1,z\neq i}^{n}\sum_{j=1}^{n}u_{zj}^{\left(k\right)}},\frac{\sum_{j=1}^{n}m_{ij}^{\left(k\right)}}{\sum_{z=1}^{n}\sum_{j=1}^{n}m_{zj}^{\left(k\right)}},\frac{u_{ij}^{\left(k\right)}}{\sum_{j=1}^{n}u_{ij}^{\left(k\right)}+\sum_{z=1,z\neq i}^{n}\sum_{j=1}^{n}l_{zj}^{\left(k\right)}})\;\\ i,j,z=1,2,\cdots ,n,\;k=1,2,\cdots ,K \end{array}$$

${\tilde{S}}_{i}^{\left(k\right)}$ represents the triangular fuzzy number of the single ranking weight of the *i* risk factor judged by the *k* expert.

It is assumed that the index layer under the target layer is the first layer, and each sub-index layer is the second ~ (n-1) layer in turn. Then, the total ranking weight relative to the target layer obtained by iterative calculation of single ranking weight of each factor can be expressed as:


(3) $$h_{i}^{\left(k\right)}=\Pi_{m=1}^{n-1}S_{i}^{\left(k)(m\right)},k=1,2,\cdots K,i=1,2,\cdots n$$

${\tilde{S}}_{i}^{\left(k)(m\right)}$ is the weight of the *m*-level index judged by the *k* expert, and $h_{i}^{\left(k\right)}$ is the total ranking weight of the bottom index to the target level.

In order to facilitate sorting and comprehensive weight calculation, the results are defuzzified. Given a triangle fuzzy number Ñ = (*l, m, u*), the defuzzification value of the triangle fuzzy number can be calculated by using Formula (4).


(4) $$\begin{array}{r} {Ñ}_{\text{defuzzification}}=\frac{l+2m+u}{4} \end{array}$$

### Fuzzy comprehensive evaluation method

The fuzzy comprehensive evaluation method is a kind of comprehensive evaluation method based on fuzzy mathematics. The fuzzy relation synthesis principle is used to quantify the factors that are relatively fuzzy and difficult to be quantified and express them with accurate mathematics. Fuzzy comprehensive evaluation is characterized by strong systematicness and clear results. It is mainly used for comprehensive evaluation of objects affected by multidimensional factors and unstructured or difficult to quantify problems. The data in this study are all qualitative data, and have the characteristics of multilevel, multi-factor, and fuzziness. Therefore, the fuzzy comprehensive evaluation method is suitable for this study. The fuzzy comprehensive evaluation method is used to evaluate the implementation status of GI EEE. The specific analysis steps are as follows.

(1) Determine the evaluation object factor Set *U* and evaluation Set *V*. At the same time, determine the weight of each influencing Factor *W*.

(2) Establish the scoring membership function and comprehensive evaluation Matrix *R* of each factor, calculate the membership Degree *R*, and obtain the fuzzy set.

(3) The fuzzy comprehensive evaluation Set *Y* = *W*×*R* is calculated by the comprehensive evaluation matrix *R*.

(4) Calculate the comprehensive evaluation score *S* = *W*×*N* of the evaluation object with the measurement Scale *N*.

### Data source

In order to obtain index weight, constructing judgment matrix is the key point of fuzzy AHP. According to the evaluation criteria in Table 2, 35 experts (teachers, managers, and scholars) in this study were given the empowerment table of evaluation indicators of GI EEE through email and an offline interview. The experts were asked to rate the relevant indicators based on their own research or experience. In order to obtain the original data of this study, the formal survey of this questionnaire selected the research objects that met the research conditions. From September to December 2021, questionnaires were distributed and collected by star in Hebei province, with 219 valid samples collected in total. Consistent with the pre-survey, the sample of this survey is still dominated by men (51.36%). Most of the respondents (86.49%) were teachers. Engineering was the highest discipline (specialty) background of the respondents (46.22%), of which digital engineering accounted for 54.36; green engineering accounted for 36.89. The teaching period is mainly 1–3 years and 4–6 years, accounting for 62.86%, which is related to the short development time of GI EEE in China. Approximately, 82.36% of the respondents have participated in teacher training, which benefited from the great attention and promotion of teacher training by the Steering Committee for Graduate Education of Engineering Specialty in recent years. Universities (66.89%) and enterprises (12.34%) were the main channels for the respondents to obtain financial aid, while only 7.63% of the respondents obtained financial aid from the government (such as educational reform projects of the education department). Only 53.96% of the respondents have carried out interdisciplinary cooperation, among which 36.94 and 28.74% have carried out interdisciplinary cooperation in education and teaching and academic research, respectively. The expertise of the respondents is very uniform.

## Results and discussion

### Determination of weight based on fuzzy AHP

According to the evaluation criteria in Table 2, more than 35 experts judged the relative importance of 21 indicators. Take the judgment results of A11–A13 indicators as an example to illustrate the calculation. The judgment results of Expert 1 are shown in Table 3.

**Table 3:** Judgment on the importance of each risk factor under meteorological conditions.

	***C*** **_1_**	**A11**	**A12**	**A13**
Expert 1	A11	(1,1,1)	(1/4,1/3,1/2)	(1/9,1/8,1/7)
	A12	(2,3,4)	(1,1,1)	(1/3,1/2,1)
	A13	(7,8,9)	(1,2,3)	(1,1,1)

According to Table 3, the fuzzy judgment matrix of this level is constructed as follows:


(5) $$\begin{array}{r} {Ã}_{C_{1}}^{\left(1\right)}=\left[\begin{array}{c} \begin{array}{ccc} \left(1,1,1\right) & \left(1/4,1/3,1/2\right) & \left(1/9,1/8,1/7\right) \end{array}\\ \begin{array}{ccc} \left(2,3,4\right) & \left(1,1,1\right) & \;\left(1/3,1/2,1\right) \end{array}\\ \begin{array}{ccc} \left(7,8,9\right) & \left(1,2,3\right) & \;\left(1,1,1\right) \end{array} \end{array}\right] \end{array}$$

Formula (2) was used to calculate the fuzzy judgment matrix ${Ã}_{C_{1}}^{\left(1\right)}$, and the triangular fuzzy number of the indicator weight vector is as follows:


(6) $${\tilde{S}}_{C_{1}}^{\left(1\right)}=\left(\begin{array}{l} {\tilde{S}}_{N_{1}}^{\left(1\right)}\\ {\tilde{S}}_{N_{2}}^{\left(1\right)}\\ {\tilde{S}}_{N_{3}}^{\left(1\right)} \end{array}\right)=\left[\begin{array}{l} \begin{array}{lll} 0.0668 & 0.0860 & 0.1175 \end{array}\\ \begin{array}{lll} 0.1854 & 0.2654 & 0.3667 \end{array}\\ \begin{array}{lll} 0.5408 & 0.6486 & 0.7347 \end{array} \end{array}\right]$$

Finally, formula (3) is used to calculate the triangular fuzzy value of each index weight, and the mean value is taken for the judgment results of each expert. Then, Formula (4) is used to defuzzify the results. This round of weight survey recovered a total of 35 scoring tables. In this study, the software Yaahp was used for auxiliary calculation, and the consistency test of each expert's grading table was carried out one by one. The arithmetic mean was used to calculate the weight of the score integration of 35 experts. The final weight calculation results of evaluation indicators at all levels are shown in Table 4.

**Table 4:** Weight of evaluation index of GI EEE.

**Main criterion layer**	**Weight 1**	**Sub-criteria layer**	**Weight 2**	**Weight 3**	**Scheme layer**	**Weight 4**	**Weight 5**
A	0.389	A1	0.304	0.118	A11	0.387	0.046
					A12	0.264	0.031
					A13	0.349	0.041
		A2	0.367	0.143	A21	0.291	0.042
					A22	0.312	0.045
					A23	0.397	0.057
		A3	0.329	0.128	A31	0.291	0.037
					A32	0.396	0.051
					A33	0.313	0.040
B	0.324	B1	0.428	0.139	B11	0.334	0.046
					B12	0.329	0.046
					B13	0.337	0.047
		B2	0.572	0.185	B21	0.296	0.055
					B22	0.383	0.071
					B23	0.321	0.059
C	0.287	C1	0.396	0.114	C11	0.326	0.037
					C12	0.347	0.039
					C13	0.327	0.037
		C2	0.604	0.173	C21	0.262	0.045
					C22	0.426	0.074
					C23	0.312	0.054

According to Table 4, the relative weights of training education, collaborative education, and situational education in the main criteria layer to the target layer are 38.9, 32.4, and 28.7%, respectively. It can be seen that the evaluation dimension with the highest weight is training education. At present, the most important education is to strengthen the ethics education of GI engineering in the process of cultivating college students. The coordination of GI EEE is becoming more and more important. Integration and co construction are important pursuits of GI EEE. Integration refers to the deep integration of GI EEE with economy and society; engineering practice and talent training EEE should be of real value to man, nature, and society. Joint construction is to expand education supply through multiple channels. The joint participation of multiple subjects provides a qualitative opportunity to promote the connotative and structured development of GI EEE. GI EEE is concrete and diverse. In the process of practice, each country not only has interoperability, but also the actual situation is very different. How to correctly grasp the development situation of international GI EEE and accurately implement policies according to the practical development trend of GI EEE has a profound impact on the effectiveness of regional GI EEE.

### The results of fuzzy comprehensive evaluation of sub-criterion layer

(1) Construct the factor set. According to Table 2, the factor set of fuzzy comprehensive evaluation of the sub-criterion layer is γ = {γ1, γ2, γ3}, where γ∈{*A*1, *A*2, *A*3, *B*1, *B*2, *C*1, *C*2}.

(2) Construct the comments set of the sub-criteria layer. This study divides the reality of the key mechanism of industrial process ethics education into five grades as follows: *V* = {*V*1, *V*2, *V*3, *V*4, *V*5}A. This means very poor, poor, average, good, and very good.

(3) Construct the sub criteria layer weight set. According to the weights of evaluation indicators at all levels, this study constructs the weight set vectors of indicators, which are as follows:


$$\begin{array}{l} WA1=\{0.387,0.264,0.349\};WA2=\{0.291,0.312,0.397\};\\ WA3=\{0.291,0.396,0.313\};WB1=\{0.334,0.329,0.337\};\\ WB2=\{0.296,0.383,0.321\};WC1=\{0.326,0.347,0.327\};\\ \;WC2=\{0.262,0.426,0.312\} \end{array}$$

The original data evaluation matrix is shown in Table 5.

**Table 5:** The original data evaluation matrix.

**Main criterion layer**	**Sub-criteria layer**	**Scheme layer**	**V1**	**V2**	**V3**	**V4**	**V5**	**Mean value**
A	A1	A11	6	36	114	52	11	3.1187
		A12	4	35	122	49	9	3.1096
		A13	9	32	98	63	17	3.2146
	A2	A21	15	32	109	55	8	3.0411
		A22	19	41	104	34	21	2.9863
		A23	8	31	113	55	12	3.1461
	A3	A31	10	34	105	61	9	3.1142
		A32	11	36	114	47	11	3.0502
		A33	13	30	109	57	10	3.0959
B	B1	B11	15	29	124	38	13	3.0228
		B12	14	34	121	40	10	2.9909
		B13	12	33	107	58	9	3.0868
	B2	B21	8	26	122	55	8	3.1324
		B22	7	28	116	53	15	3.1872
		B23	9	24	113	60	13	3.2009
C	C1	C11	15	33	120	42	9	2.9863
		C12	14	31	117	47	10	3.0365
		C13	5	29	128	46	11	3.1324
	C2	C21	11	38	123	38	9	2.9817
		C22	12	31	126	42	8	3.0137
		C23	14	29	118	48	10	3.0502

According to Table 5, the A1 membership matrix of key education status evaluation of GI EEE is constructed as follows:


$$\begin{array}{l} RA1=\left[\begin{array}{c} \begin{array}{ccccc} 0.0274 & 0.1644 & 0.5205 & 0.2374 & 0.0502 \end{array}\\ \begin{array}{ccccc} 0.0183 & 0.1598 & 0.5571 & 0.2237 & 0.0411 \end{array}\\ \begin{array}{ccccc} 0.0411 & 0.1461 & 0.4475 & 0.2877 & 0.0776 \end{array} \end{array}\right] \end{array}$$

The compound operation results of fuzzy matrix are as follows:


$$\begin{array}{l} YA1=WA1*RA1\\ =\{0.387,0.264,0.349\}*\\ \left[\begin{array}{l} \begin{array}{rrrrr} 0.0274 & 0.1644 & 0.5205 & 0.2374 & 0.0502 \end{array}\\ \begin{array}{rrrrr} 0.0183 & 0.1598 & 0.5571 & 0.2237 & 0.0411 \end{array}\\ \begin{array}{rrrrr} 0.0411 & 0.1461 & 0.4475 & 0.2877 & 0.0776 \end{array} \end{array}\right]\;\\ =\left\{\begin{array}{rrrrr} 0.0298 & 0.1568 & 0.5047 & 0.2514 & 0.0574 \end{array}\right\} \end{array}$$

Similarly, fuzzy comprehensive evaluation results of other INDICATORS A2, A3, B1, B2, C1, and C2 can be obtained as follows:


$$\begin{array}{l} YA2=\left\{\begin{array}{ccccc} 0.0622 & 0.1554 & 0.4981 & 0.2258 & 0.0586 \end{array}\right\}\\ YA3=\left\{\begin{array}{ccccc} 0.0516 & 0.1513 & 0.4967 & 0.2553 & 0.0451 \end{array}\right\}\\ YB1=\left\{\begin{array}{ccccc} 0.0625 & 0.1448 & 0.5355 & 0.2078 & 0.0494 \end{array}\right\}\\ YB2=\left\{\begin{array}{ccccc} 0.0369 & 0.1179 & 0.5355 & 0.2567 & 0.0529 \end{array}\right\}\\ YC1=\left\{\begin{array}{ccccc} 0.0514 & 0.1419 & 0.5571 & 0.2042 & 0.0455 \end{array}\right\}\\ YC2=\left\{\begin{array}{ccccc} 0.0562 & 0.1507 & 0.5573 & 0.1943 & 0.0415 \end{array}\right\} \end{array}$$

According to the maximum membership degree principle, the maximum membership degree values in the sub-criterion layer are all general, wherein the maximum membership degrees of A1, A2, A3, B1, B2, C1, and C2 are 0.5047, 0.4981, 0.4967, 0.5355, 0.5355, 0.5571, and 0.5573, respectively. Therefore, it is judged that the performance of GI EEE in this area is at the average level.

### The results of fuzzy comprehensive evaluation of main criterion layer

According to the membership evaluation results of A1, A2, A3, B1, B2, C1, and C2 obtained above, the fuzzy comprehensive evaluation value of the winner criterion layer can be obtained. The evaluation results of the membership degree of *A* = {*A*1, *A*2, *A*3} training education are as follows:


$$\begin{array}{l} YA=WA*RA\\ =\left\{\begin{array}{rrr} 0.304 & 0.367 & 0.329 \end{array}\right\}*\\ \left[\begin{array}{l} \begin{array}{rrrrr} 0.0298 & 0.1568 & 0.5047 & 0.2514 & 0.0574 \end{array}\\ \begin{array}{rrrrr} 0.0622 & 0.1554 & 0.4981 & 0.2258 & 0.0586 \end{array}\\ \begin{array}{rrrrr} 0.0516 & 0.1513 & 0.4967 & 0.2553 & 0.0451 \end{array} \end{array}\right]\;\\ =\left\{\begin{array}{lllll} 0.0489 & 0.1545 & 0.4996 & 0.2433 & 0.0538 \end{array}\right\} \end{array}$$

$$\begin{array}{l} YB=WB*RB\\ =\left\{\begin{array}{rr} 0.428 & 0.572 \end{array}\right\}*\left[\begin{array}{l} \begin{array}{rrrrr} 0.0625 & 0.1448 & 0.5355 & 0.2078 & 0.0494 \end{array}\\ \begin{array}{rrrrr} 0.0369 & 0.1179 & 0.5355 & 0.2567 & 0.0529 \end{array} \end{array}\right]\;\\ =\left\{\begin{array}{lllll} 0.0479 & 0.1294 & 0.5355 & 0.2358 & 0.0514 \end{array}\right\} \end{array}$$

$$\begin{array}{l} YC=WC*RC\\ =\left\{\begin{array}{rr} 0.396 & 0.604 \end{array}\right\}*\left[\begin{array}{l} \begin{array}{rrrrr} 0.0514 & 0.1419 & 0.5571 & 0.2042 & 0.0455 \end{array}\\ \begin{array}{rrrrr} 0.0562 & 0.1507 & 0.5573 & 0.1943 & 0.0415 \end{array} \end{array}\right]\;\\ =\left\{\begin{array}{lllll} 0.0543 & 0.1472 & 0.5572 & 0.1982 & 0.0431 \end{array}\right\} \end{array}$$

According to the principle of maximum degree of membership, the maximum degree of membership in the main criterion layer is general, among which the maximum degrees of membership of A, B, and C are 0.4996, 0.5355, and 0.5572, respectively. Therefore, it is judged that the development of GI EEE in this region is at the general level in terms of cultivation education, collaborative education, and situational education.

### The results of fuzzy comprehensive evaluation of target layer

According to *G* = {*A, B, C*} obtained above, the final evaluation results of the membership degree of the target layer can be obtained as follows:


$$\begin{array}{l} YG=WG*RG\\ =\left\{\text{​}\begin{array}{rrr} 0.389 & 0.324 & 0.287 \end{array}\text{​}\right\}*\left[\text{​​}\begin{array}{l} \begin{array}{rrrrr} 0.0489 & 0.1545 & 0.4996 & 0.2433 & 0.0538 \end{array}\\ \begin{array}{rrrrr} 0.0479 & 0.1294 & 0.5355 & 0.2358 & 0.0514 \end{array}\\ \begin{array}{rrrrr} 0.0543 & 0.1472 & 0.5572 & 0.1982 & 0.0431 \end{array} \end{array}\text{​​}\right]\;\\ =\left\{\begin{array}{lllll} 0.0543 & 0.1472 & 0.5278 & 0.2279 & 0.0500 \end{array}\right\} \end{array}$$

According to the principle of maximum membership degree, the membership degree of general was the highest (0.5278). Therefore, it is judged that the implementation status of the key education of GI EEE in Hebei is at the general level.

### Discussion

From the above research, it can be seen that the maximum membership degree of the implementation status of key education in GI EEE is average. In order to further distinguish the performance differences of each indicator, this study tries to calculate the individual and overall scores of each indicator, which is conducive to reflect the evaluation results to the maximum and more truly and effectively. In this study, each evaluation element of the comment set is assigned, with a value of 50, 60, 70, 80, and 90, respectively. Thus, the semantic scale of subjective evaluation is quantified. Table 6 shows the evaluation scores of various indicators on the implementation status of key education of GI EEE after analyzing the scoring data of 219 experts. A score of 80 or above is considered excellent, 70–80 is considered good, 60–70 is considered acceptable, and below 60 is considered unqualified.

**Table 6:** The scores of various indicators on the implementation status of key education of GI EEE.

**Target layer**	**Sub-criteria layer**	**Score**	**Sub-criteria layer**	**Score**	**Scheme layer serial**	**Score**
Implementation status of key education in GI EEE (Score: 70.8368)	Training and education A	70.9699	Faculty A1	71.4978	Teachers' professional level and ability A11	71.1872
					Number and structure of teaching staff A12	71.0959
					Teacher career development opportunities A13	72.1461
			Teaching process A2	70.6570*	Course material A21	70.4110*
					Teaching case A22	69.8630*
					Practice resource A23	71.4612
			Teaching quality A3	70.8313	Combination of theory and practice A31	71.1416
					Full cycle ethics education A32	70.5023*
					Course evaluation and improvement A33	70.9589
	Collaborative education B	71.1482	Government B1	70.3386*	Supporting system construction B11	70.2283*
					Attention at the policy level B12	69.9087*
					Funding level B13	70.8676
			Enterprise organization B2	71.7539	Attach importance to talent ethical literacy B21	71.3242
					Participation form and quality B22	71.8721
					Willingness to participate B23	72.0091
	Situational education C	70.3050*	International vision C1	70.5151*	Grasp of Global Frontier Situation C11	69.8630*
					Academic dialogue C12	70.3653*
					Practice dialogue C13	71.3242
			Regional discourse C2	70.1672*	Theoretical localization C21	69.8174*
					Practice localization C22	70.1370*
					Local demands capture C23	70.5023*
	Mean value	70.8077	Mean value	70.8230	Mean value	70.8089

* means below average.

Based on the statistical empirical test, this paper makes a factual judgment on the implementation status of the key mechanism of GI EEE in Hebei province.

(1) Analysis of key education. According to the score of evaluation index, the implementation status of key education of GI EEE is good and inferior. It is only 0.8368 higher than the good baseline, and there is still room for improvement. Among them, cultivation education (70.9699) and collaborative education (71.1482) are above the average level, while situational education (70.3050) is relatively low. According to the weight distribution of evaluation indexes, cultivation education has the highest weight and the most prominent importance. The second is collaborative education and cultivation education, but the evaluation results are contrary to them. It shows that there is a gap between theory and practice in GI EEE. The main reason lies in the late start of GI EEE. For a long period of time, the focus of discussion is how to do the university itself, and the attention to localization and coordination issues is obviously insufficient.

(2) Analysis of key situations. In terms of situational education, there was no significant difference in the scores of GI EEE in an international perspective (70.5151) and regional discourse (70.1672). The key direction of promoting the internationalization of GI EEE from an international perspective is to actively carry out educational dialogues, including grasping the global frontier situation and theory localization. From the perspective of reality, the process of localization of GI EEE needs a long time to accumulate and cannot be accomplished overnight. Only step by step and in accordance with the world's leading trends can we effectively improve the local meaning of GI EEE.

(3) Analysis of key directions. Combined with the score data of each index of the sub-criteria layer and the program layer, the score of each index of the subordinate of collaborative education is basically good. Among them, the score of attaching importance to policy was the lowest, only 69.9087. This reflects that the government's willingness to participate in the ethical education of GI engineering is not enough, especially the ethical literacy of talents has not been widely valued by the government. It is not conducive to spreading a belief in the usefulness of ethics throughout society. On the whole, there are still some barriers in the coordination of GI EEE. No matter the engineering government, enterprises, college teachers, and engineering students, all have a non-committal attitude toward the implementation of GI EEE.

(4) Analysis of key points. In the strategy of GI EEE, the educational idea is the cornerstone, the teaching is the difficulty, and the teaching staff is the support. Returning to the level of colleges and universities, the overall performance of the indicators of training and education subordinates is better. The teaching staff (71.4978), teaching process (70.6570), and teaching quality (70.8313) in the sub-criteria were all at a good level. According to the score of the program level, the optimized space of training education includes teaching cases, full-cycle ethics education, etc.

In view of the above analysis, the following countermeasures are put forward.

(1) The dynamic balance of training and education on the connection between supply and demand. The cultivation education in the key education of GI EEE is generated from the mutual construction of educational ideas, course teaching, and teachers. Therefore, the pursuit of high-quality development of GI EEE should also closely focus on the above three factors. According to the practical needs, the vitality and activity of GI EEE should be continuously enhanced. This needs to strengthen the effect of educational reform and innovation, anchor the basic aspect of the educational concept, pay attention to the basic points of teaching, and optimize the supporting line of the teaching staff. It is helpful to improve the quality and the level of GI EEE. The idea is the forerunner of action, the deviation between educational ideas and practice orientation will directly affect the effect of education. Only when the rational understanding of EEE is in place, the action can be targeted. The educational idea of GI engineering ethics is to adapt to the forefront changes of engineering ethics teaching and learning. This should follow the law of students' cognitive development, growth and talent, teaching and educating, with student-centered, interdisciplinary, and collaborative education in the first place. The practice orientation of GI EEE and even the whole higher education emphasizes that the roles and functions of teachers and students should be changed positively in educational practice. As a cross-body of multi-disciplines, GI engineering ethics needs to guide the development of GI EEE from the cross-disciplinary perspective. In terms of horizontal interconnection, orderly infiltration of various elements, integration and mobilization of multi-subjects and all-round coordinated participation should be realized. This can build a good development platform for the high-quality development of GI EEE. GI EEE is expanded to practice, and the theory and practice are mapped to each other. Alumni engineers participate in classes and give full play to their advantages of mentoring and form good positive feedback in students' minds. The ability and accomplishment of full-time teachers can be improved by opening open classes, guiding young teachers by key teachers, and establishing engineering ethics teacher development centers.

(2) The governance means of GI EEE. Construct the collaborative system of GI EEE and actively integrate various forces. This is conducive to promoting the transformation of colleges and universities from fighting alone to multi-subject coordinated development, and fully fermenting the power of high-quality development. A reasonable and effective system supply criterion is the value dimension to maintain and promote the coordinated and sustainable development of GI EEE. In terms of policy, the urgency and significance of GI EEE should be realized from the inevitable trend of the development of global EEE. The ethics education of GI engineering is a complex system engineering involving a wide range and far-reaching influence. It plays an important role in the improvement of higher engineering education system and the development of engineering practice in China. At the same time, the complexity and global nature of modern engineering make the responsibilities of engineering talents more extensive than ever before. Engineering students need to be prepared for their future career development. Funding resources and teaching practice resources are the two most urgently needed resources for GI EEE. The development of GI EEE is highly dependent on scientific research and teaching funds from the government, forming an engineering ethics funding system dominated by educational reform projects, supplemented by self-science and social science funds. In this way, a number of high-level scientific research projects and achievements can be generated and turned into GI EEE and teaching resources. The key reason for the long-term disconnection between the theoretical teaching and practical teaching of GI EEE is the lack of practical resources. Universities and enterprises should make full use of the existing industry-university cooperation platform, off-campus cooperative practice teaching bases, off-campus practice training sites, etc., to move the practice teaching link of engineering ethics to the practice site of enterprises. This will help students understand the technical factors, human factors, and economic factors involved in GI engineering in the real world, and have a more real sense of abstract concepts, such as safety, risk, cost, and efficiency.

(3) The ethical situational education of GI engineering. Situational education is the most important key education. How to realize the coordination between international vision and regional discourse in the development of GI EEE has become a key issue to be considered urgently. To construct the Chinese discourse of GI EEE, we need to absorb the latest, scientific, and advanced achievements from the world and take the lead in the development of The Times. In designing the subject system, teaching system, teaching material system, and management system, it is necessary to pay attention to absorbing new educational ideas and scientific teaching methods. It is necessary to make new progress in practical cooperation and broaden the space and object of GI EEE. In essence, the ethical education of GI engineering is the ideological work of human beings. It is necessary to re-understand the ethics education of GI engineering from the height of ideological and political education. Colleges and universities will bring GI EEE into the big ideological and political pattern, and further improve the status of GI EEE. In the course design, course teaching, and other links, professional course teachers dig deeply into the ethical norms, value orientation, and engineering spirit contained in professional courses. In addition, GI engineering ethics teachers can show the mission of Chinese engineers with the help of fresh materials of “technological anti-epidemic” in the course design.

## Conclusions

Based on the key mechanism of GI EEE proposed above, the initial evaluation index system of the implementation status of the key mechanism of GI EEE is constructed. Firstly, the fuzzy analytic hierarchy process is used to assign weights to each index based on 35 expert weights. It can be found that the importance of different evaluation levels and indicators for EEE is different, and all evaluation levels and indicators maintain a dynamic balance. The importance of the cultivation mechanism was the highest, while the importance of the collaborative mechanism and the situational mechanism decreased in descending order. Secondly, based on 219 evaluation samples, the fuzzy comprehensive evaluation method is used to empirically evaluate the implementation status of key mechanisms of GI EEE. Finally, on the basis of the evaluation, the fact-oriented objective judgment is made on the key mechanism of GI EEE.

The results of this study are as follows. (1) The key education of GI EEE includes cultivation education of micro dimension, collaborative education of medium dimension, and situational education of macro dimension. The micro dimension of training education is to enhance the quality of the supply of higher education. Meso-dimension collaborative education is the collaborative power of ethical education. Macro-dimension situational education is the field space of developing regional context. (2) The factor affecting the ethical education of GI engineering is cultivation education. At present, the most important education is to strengthen the education of GI engineering ethics in the training process of college students. The coordination of GI EEE is becoming more and more important, and the integration and construction are the important pursuit of GI EEE. (3) The ethical education of GI engineering is at a general level in the development of cultivation education, collaborative education, and situational education. The implementation status of the key education of GI EEE is a good deviation, and there is still a large space for improvement. The key direction of promoting the internationalization of GI EEE from an international perspective is to actively carry out educational dialogues, including grasping the global frontier situation and theory localization. The government's willingness to participate in the ethical education of GI engineering is not enough, especially the ethical literacy of talents has not been widely valued by the government. The optimized space of training education includes teaching cases and full-cycle ethical education. (4) Realize the dynamic balance of training and education on the connection between supply and demand. Enrich the governance means of GI EEE. Deepen the ethical situational education of GI engineering.

Although the purpose of this study has been achieved, there are still some issues that need to be studied in the future. One is the lack of dynamic changes in evaluation data. Future studies can consider the dynamic changes of key variables in time series so as to accurately grasp the evolution trend. The second is about the sample structure. In the survey samples, teachers and scholars are in the majority. The small sample size of engineering community managers may affect the conceptual validity of the survey results to some extent. Future studies should increase the sample size of engineering community managers.

## Data availability statement

The data presented in this study are available on request from the corresponding author.

## Author contributions

SY and NZ: conceptualization and validation. SY: methodology, software, and writing—review and editing. NZ: writing—original draft preparation. Both authors have read and agreed to the published version of the manuscript.

## Funding

This research was funded by the 11th Batch of Teaching Research Projects of Hebei Agricultural University in 2021, Research on Interdisciplinary Cultivation and Reading Practice Teaching Mode of Agricultural College Students (2021C-39), the Construction of Ideological and Political Case Base of Organizational Behavior (2021B-2-01), the Project of the Chinese Association of Degree and Graduate Education, Research on the Cultivation of Practical Ability of Graduate Students Majoring in Agronomy (2020MSB37), and Hebei Agricultural University First-Class Undergraduate Course Construction Project Management System Engineering.

## Conflict of interest

The authors declare that the research was conducted in the absence of any commercial or financial relationships that could be construed as a potential conflict of interest.

## Publisher's note

All claims expressed in this article are solely those of the authors and do not necessarily represent those of their affiliated organizations, or those of the publisher, the editors and the reviewers. Any product that may be evaluated in this article, or claim that may be made by its manufacturer, is not guaranteed or endorsed by the publisher.
